# CAN SENSORY DISTURBANCES DUE TO INJURY TO THE INFRAPATELLAR BRANCH OF THE SAPHENOUS NERVE BE PREVENTED BY AN OBLIQUE INCISION?

**DOI:** 10.1590/1413-785220243204e277962

**Published:** 2024-10-07

**Authors:** Julio Cesar Gali, Rodrigo de Souza Holtz, Marcello Scimini Lepispico, Enzo Barrio, João Otavyo Pereira Le Senechal, Julio Cesar Gali

**Affiliations:** 1. Pontificia Universidade Paulista, Faculdade de Ciencias Medicas, Sorocaba, SP, Brazil.; 2. Pontificia Universidade Paulista, Faculdade de Ciencias Medicas, Nucleo de Ortopedia e Traumatologia Esportiva, Sao Paulo, SP, Brazil.

**Keywords:** Anterior Cruciate Ligament Reconstruction, Nervous tissue injuries, Hamstring Muscle Tendons, Reconstrução do ligamento cruzado anterior, Tecido nervoso lesões, Tendões dos Músculos Isquiotibiais

## Abstract

**Objective:**

To evaluate the incidence of injuries to the infrapatellar branch of the saphenous nerve (IPBSN) after anterior cruciate ligament reconstruction (ACLR) with an oblique incision for hamstring graft harvesting.

**Methods:**

In total, 59 knees (from 57 patients) were evaluated in the follow-up of ACLR for six months. We drew a horizontal line parallel to the ground, passing through the most medial portion of the surgical incision and another, perpendicular to the first, starting at the tibial tuberosity (TT). We measured the length and angle of the cut, the distances from its most medial point to the perpendicular line, and from the TT to the horizontal line. Skin sensitivity was tested with a brush and the altered sensitivity area was measured. Patients were asked about difficulties in activities daily of living (ADL).

**Results:**

A total of 27 knees (45.7%) had sensory disorders, which persisted until the sixth postoperative month in 92.6% of them. The ADL were compromised in one knee (3.7%). No significant differences were found between the groups with and without changes in sensitivity regarding age, affected side, incision angle, or measured distances. The incision size was larger in the group without alteration in sensitivity.

**Conclusions:**

An oblique incision did not avoid IPBSN injuries. This condition rarely compromised the ADL. ***Level of Evidence II, Lesser Quality Prospective Study.***

## RESUMO

**Objetivo:**

Avaliar a incidência de lesões do ramo infrapatelar do nervo safeno (RIPNS) na reconstrução do ligamento cruzado anterior (RLCA), com incisão oblíqua para a coleta do enxerto dos isquiotibiais.

**Métodos:**

59 joelhos (57 pacientes) foram avaliados no pós-operatório da RCLA, por seis meses. Traçamos uma linha horizontal paralela ao solo, passando pela porção mais medial da incisão cirúrgica, e outra perpendicular à esta, iniciando na tuberosidade tibial (TT). Medimos o comprimento e a angulação do corte, as distâncias do ponto mais medial do corte à linha perpendicular e outra, da TT, à linha horizontal. A sensibilidade da pele foi testada com um pincel, e a área alterada foi mensurada. Os pacientes foram questionados sobre as dificuldades nas atividades diárias da vida (ADV).

**Resultados:**

27 joelhos (45,7%) apresentaram distúrbios sensitivos, persistentes até o sexto mês pós-operatório em 92,6% deles. As ADV foram comprometidas em um joelho (3,7%). Não houve diferença significante entre os grupos com e sem alterações da sensibilidade, relativamente à idade, ao lado comprometido, ao ângulo da incisão ou às distâncias medidas. O tamanho da incisão foi maior no grupo sem alteração de sensibilidade.

**Conclusões:**

Uma incisão oblíqua não evitou lesões no RIPNS. Essa condição raramente comprometeu as ADV. ***Nível de Evidência II, Estudo Prospectivo de Menor Qualidade.***

## INTRODUCTION

 The incidence and prevalence of anterior cruciate ligament (ACL) reconstruction (ACLR) has increased significantly in recent years, especially in women. ^1^^,^^2^ Still, primary ACLR are more common in men under 30 years of age, and soccer is the sport most often linked to the rupture of this ligament. ^3^ A survey by the ACL Study Group showed the evolution of the graft choice for ACLR over the last three decades. ^4^ In 1992, the bone-patellar tendon-bone graft was chosen for primary ACLR by about 90% of the surgeons surveyed. However, the preference for hamstring tendon grafts increased during the study period and, in 2020, flexor tendon grafts were reportedly preferred by more than 50% of those surveyed. 

 Bertram et al. 5 were the first to describe a case of infrapatellar branch of the saphenous nerve (IPBSN) neuralgia due to entrapment of the nerve branch by scar tissue. Due to its anatomical location, there is a potential risk of injury to the IPBSN during the removal of the flexor tendons for ACLR. Anatomically, the IPBSN shows an average tilt angle of 17.5°± 6.1° ^6^ and, theoretically, an oblique incision would exhibit a lower risk of injuring this nerve branch than a vertical incision. In another anatomical study, Wisbech Vange et al. ^7^ showed that a diagonal incision can reduce risk of lesions in this branch. In fact, several authors have published that the oblique incision holds a lower risk of IPBSN injury. ^8^^-^^18^

 On the other hand, the method of evaluating skin sensitivity related to IPBSN injury is variable; some authors have used the touch of a needle, ^9^^,^^11^^,^^14^^,^^17^^,^^18^ others, digital pressure measurements, ^10^ self-reported skin sensitivity, ^8^ or questionnaires. ^19^ We believe it is important to perform skin sensitivity assessments with an atraumatic approach and prospective follow-up. This study aimed to evaluate whether using an oblique incision could prevent IPBSN injury during ACLR with hamstring tendons. 

## MATERIALS AND METHODS

A total of 59 knees from 57 patients were evaluated in the postoperative period of ACLR with hamstring tendon grafts, collected via oblique incision, for six months. All patients signed an informed consent form to participate in this study, which was approved by the Research Ethics Committee of the institution, under number 33453220.3.0000.5373. Patients with multiple ligament injuries, ACLR revisions, and those who had already undergone any type of previous surgical procedure in the knee region under study, as well as patients with any peripheral neurological abnormality, were excluded from this study. Patients diagnosed with anterior cruciate ligament injury by clinical examination and confirmation by magnetic resonance imaging were included. All patients were operated by the senior occupational surgeon.

 In total, 52 patients were male (91.2%) and five (8.7%) were female; the age of patients ranged from 14 to 75 years [mean of 34.4 years and standard deviation (SD) of ± 11.2 years]; and 31 knees (52.5%) were right and 28 (47.4%) were left. Oblique incision was performed to remove hamstring tendons, in parallel to the IPBSN path and with blunt dissection. During this procedure, the knees were flexed and the hips externally rotated to reduce tension on the saphenous nerve. ^20^ This study did not aim to identify the IPBSN. The lateral femoral condyle was visualized through the anteromedial portal (AMP) created with a vertical incision. The femoral tunnels were then made using an accessory anteromedial portal (AAMP), also positioned with a vertical, distal, and medial incision relative to the AMP, with the knee flexed at 90°. 

**Figure 1.**
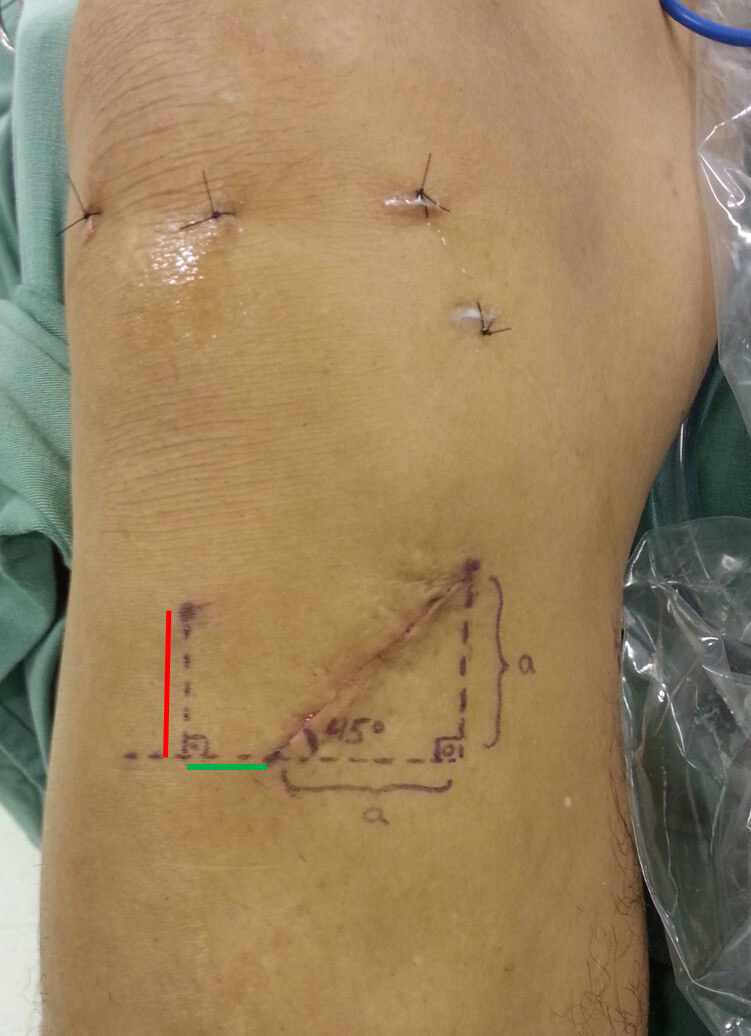
Frontal view of a right knee showing the incision and its angle, the mediolateral distance, in green, and the craniocaudal, in red.

 After closing the subcutaneous tissue, a horizontal line was drawn parallel to the ground using a sterile marker pen, passing through the most medial portion of the surgical incision. Another line was drawn perpendicular to this, starting at the tibial tuberosity (TT) and extending distally to meet the horizontal line ( Figure 1 ). An aseptic millimeter ruler was used to measure the length of the incision and the distance from the TT to the horizontal line, termed the craniocaudal distance. Additionally, the distance from the most medial point of the incision to the perpendicular line was measured, referred to as the mediolateral distance. Intraoperative measurements were performed by the surgeon and an assistant, who agreed on the obtained values. The “Measure Angle” tool, available on Windows, was used to obtain the cut angles from the images taken during surgeries. 

Follow-up was performed one, two, three, and six months postoperatively. During consultations, skin sensitivity around the incision was assessed by the same examiner, with a soft monofilament brush. Initially, the brush was applied to the patients’ hands, allowing them to recognize the sensory stimulus; then, the skin sensitivity assessment around the incision was conducted with the patients with their eyes closed. Thermal and pain sensitivities were not assessed.

 The boundaries of the area with altered sensitivity were marked with a sterile pen on the points of sensitivity change based on reports by the patients at intervals of 1.5 cm, thus creating a sensory map. The areas were photographed, then measured in ^2^ using the Photoshop® software (Adobe Inc., USA). At follow-up, the patients were asked about the presence of discomfort when kneeling, alteration in knee function, and difficulty in performing activities of daily living. 

For the statistical analysis, the Mann-Whitney test was applied to compare the groups with and without sensory alteration, in relation to age, angle, and size of the incision, as well as craniocaudal and mediolateral distances. The Fisher’s test was used to compare female and male patients, and the Chi-square test was used to compare the presence of alterations observed on the right and left side. In all tests, the index of significance (α) was set at 0.05 or 5%.

## RESULTS

The mean size of the incisions was 38.7 mm, ranging from 30.0 to 50.0 mm, and SD was ± 5.20 mm; the mean angle of the cuts was 30 mm (12 to 45 mm; SD ± 6.8 mm), the mean mediolateral distance was 17.6 mm (0.0 to 30.0 mm; SD ± 6.9 mm), and the mean craniocaudal distance was 16.9 mm (0.0 to 40.0 mm; SD ± 9.6 mm). Changes in sensitivity were found in 27 knees, from 27 patients (45.7%); 32 knees did not present different sensory perception (54.2%). The two patients with bilateral ACLR had no changes in sensitivity in either knee. In the comparison between the groups with and without cutaneous dysesthesia, no significant differences were observed in relation to the age of the patients (p = 0.52), affected side (p = 0.3223), or angle of the incision (p = 0.18). The groups also did not differ significantly in relation to craniocaudal distance (p = 0.4038). However, when compared in terms of the mediolateral range, the results suggest higher values for the group with no change in sensitivity (p = 0.0592), even though they did not reach the significance level (0.05).

The presence of skin sensitivity alteration in females occurred in all cases (100%) and was significantly higher than that observed in males. The size of incision in the group without sensitivity alteration was significantly larger than in the group of patients who presented sensitivity alteration (p = 0.0430). However, the mean incision size in women was 36.0 mm, being below the mean of the group with sensory alterations, which was 38.7 mm. In two knees (7.4%), the paresthesia observed in the first month disappeared in subsequent assessments, in the second and third month of follow-up each. In the other knees, the change in sensitivity persisted in the evaluation six months after surgery. Only one of the knees (3.7%) had functional impairment due to neurological disorder, according to the patient.

 In our sample, the mean area of diverse perception in the knees with altered sensitivity was 20.7 ^2^ . The incidence of sensory neurological disturbance in the region lateral to the TT occurred in 88.8% of knees. The percentage of alterations in cutaneous perception in the region proximal to the TT was 37.0%, while in the region distal to this bony prominence, it was 62.9%. On the medial side, sensory disturbance occurred in 11.1% of the knees with sensory alteration ( Table 1 ). Table 2 provides further information on age, sex, knee side, size and angulation of incisions, and craniocaudal and mediolateral distances obtained from patients without changes in skin sensitivity. Table 3 shows information on patients with altered sensitivity plus the affected area. 

**Table 1.** Area of tactile sensitivity alteration

Compromised area	Proximal to TT	Distal to TT
Medial to TT	2 (7.4%)	1 (3.7%)
Lateral to TT	8 (29.6%)	16 (59.2%)

**Table 2.** Information regarding age, sex, knee side, size and angulation of incisions, and craniocaudal and mediolateral distances obtained from patients without changes in skin sensitivity

	Age	Side	Sex	Distances (mm)		Incision Size (mm)	Incision Angle (degrees)
				Craniocaudal	Mediolateral		
1	42	R	M	13	20	40	37
2	40	R	M	13	18	45	22
3	24	L	M	30	13	48	32
4	26	R	M	10	24	40	37
5	45	R	M	28	30	40	29
6	34	L	M	15	7	45	30
7	46	R	M	25	25	45	28
8	19	L	M	25	12	36	39
9	34	L	M	0	25	30	34
10	46	R	M	40	20	45	34
11	47	R	M	20	30	40	36
12	17	R	M	6	16	40	35
13	35	R	M	5	20	32	26
14	22	R	M	10	20	45	17
15	32	R	M	5	20	35	19
16	33	L	M	20	25	40	36
17	41	R	M	30	20	35	24
18	35	R	M	5	20	35	36
19	32	L	M	30	15	40	27
20	34	L	M	15	10	50	26
21	42	R	M	20	25	45	25
22	42	L	M	30	10	35	36
23	25	R	M	15	25	40	34
24	23	L	M	10	10	35	35
25	25	R	M	24	25	38	30
26	50	R	M	15	25	45	36
27	38	L	M	15	15	40	28
28	35	L	M	5	30	45	36
29	44	L	M	10	10	40	32
30	14	R	M	5	15	40	31
31	43	R	M	15	20	35	27
32	43	L	M	35	15	45	32
	34.6	R	M	17.0	19.2	40.3	30.8
	9.7	19 (59.3%)	32 (100%)	10.2	6.4	4.8	5.6
	50.0	L	F	40.0	30.0	50.0	39.0
	14.0	13 (40.6%)	0 (0%)	0.0	7.0	30.0	17.0

**Table 3.** Information regarding age, sex, knee side, size and angulation of incisions, craniocaudal and mediolateral distances, and altered sensitivity area obtained from patients with sensory disorders

	Age	Side	Sex	Distances (mm)		Incision Size (mm)	Incision Angle (degrees)	Altered Sensitivity Area (cm^2^)
				Craniocaudal	Mediolateral			
1	21	L	M	20	7	43	31	4
2	41	R	M	22	13	45	45	3
3	37	L	M	25	0	50	29	48
4	31	L	M	20	17	34	44	12
5	53	L	F	30	4	40	36	42
6	34	R	M	15	25	40	33	2.25
7	14	L	M	30	10	35	42	2
8	40	R	M	11	20	33	35	11.25
9	43	R	M	20	30	30	29	30
10	34	L	M	0	15	35	35	30
11	37	L	M	25	15	40	34	30
12	26	L	F	10	10	35	19	37.5
13	32	L	F	6	16	40	23	33.25
14	46	R	M	20	15	40	23	24
15	24	R	M	5	20	45	26	12.7
16	27	R	F	20	20	30	24	18
17	75	R	M	15	20	30	27	14
18	38	L	M	10	20	45	24	54
19	22	L	M	16	15	35	26	40
20	41	R	M	35	30	40	27	6.25
21	24	L	F	10	10	35	30	7.5
22	23	R	M	20	20	40	27	22.5
23	37	L	M	20	5	40	26	10
24	50	L	M	25	15	35	30	10
25	33	L	M	5	20	35	35	33
26	26	R	M	20	15	35	12	6.25
27	17	R	M	0	15	30	13	16
	34.3	R	M	16.9	15.6	37.6	29.1	20.7
	12.7	12 (44.4%)	22 (81.4%)	9.0	7.1	5.2	8.0	15.1
	75.0	L	F	35.0	30.0	50.0	45.0	54.0
	14.0	15 (55.5%)	5 (18.5%)	0.0	0.0	30.0	12.0	2.0

## DISCUSSION

The main finding of our study was that an oblique incision could not prevent changes in skin sensitivity caused by IPBSN lesions. Moreover, sensory disturbances occurred in 45.7% of the knees and persisted in 92.6% of them until the sixth month of follow-up. Despite this, discomfort when kneeling and difficulties in daily activities occurred in only one of the operated knees (3.7%).

 Other authors who assessed the use of oblique incision found that the IPBSN lesion occurred within 24% and 61.3% of the cases, with a mean of 37.5 ± 13.9% among their results. ^8^^-^^11^^,^^14^^,^^17^^,^^18^^,^^21^^,^^22^ A possible explanation for this disparity may be the method used to assess sensory disorders. 

 Luo et al. ^8^ asked their patients to demarcate the altered sensitivity area and Mirzatolooei et al. ^21^ sent a questionnaire with a diagram for the same purpose, both studies presenting very subjective methods. Sabat et al. ^9^ and Sharaby et al. ^17^ used a blunt pin; Sipahioglu et al. ^11^ used a blunt needle; and Leite et al. ^10^ performed the assessments via digital pressure. These methods were also found to be inaccurate for measuring skin sensory changes. Mousavi et al. ^14^ and Keyhani et al. ^18^ used a needle for their assessments, which can be dangerous for disease transmission. 

 We found few articles that reported complaints of neurologically injured individuals. Sabat et al. ^9^ reported that 13.5% of their cases had subjective complaints of sensitivity loss, whereas Mousavi et al. ^14^ reported that four individuals (5%) complained of pain. On the other hand, Keyhani et al. ^18^ reported that three patients (6.2%) reported pain at the incision site, without interfering with their activities of daily living. 

 In our sample, the mean size of the incisions was larger than those of other researchers, whose mean length ranged from 27 to 38 mm, with a mean of 33.2 ± 4.3 mm. ^8^^-^^11^^,^^14^^,^^17^^,^^18^^,^^21^^,^^22^ We noticed that larger incisions were associated with normal sensitivity. This result is contrary to what has been published by other authors. Sharaby et al. ^17^ found no differences between incision size and sensory loss; Mousavi et al. ^14^ found a correlation between the patients’ complaints and incision size; and Pękala et al. ^13^ , in a systematic review, recommended that the incision should be as small as possible to avoid IPBSN injuries. 

Sensory disturbances occurred in all women in our sample, who had a mean incision size of 36.0 mm, below the mean of 38.7 mm in the general population. We found no publications addressing this datum to compare with our results.

 When comparing our results on the altered sensitivity area with those published by other authors, we noticed a great disparity. The article by Inderhaug et al., ^23^ showed a much larger area (69 cm^2^) , whereas a smaller area was found by Luo et al. ^8^ (8.4 cm^2^). Once again, the probable explanation for the difference was the method used for the sensitivity test. In the study by Inderhaug et al. ^24^ , sensory disturbance was assessed using light touch; Luo et al. ^8^ requested patients to mark the area; and Sabat et al. ^9^ and Sipahioglu et al. ^11^ used a blunt needle. On the other hand, Mousavi et al., ^14^ as well as Keyhani et al. ^18^ employed the needle test. In our sample, the incidence of sensory alteration occurred in the region lateral to the TT in 88.8% of the knees. The descriptions found in the literature are varied; however, as a common denominator, they compromised the region lateral to the TT. ^9^^-^^11^^,^^18^^,^^19^^,^^21^^,^^22^

 Sensory alteration in the assessment six months after surgery remained in 92.6% of the knees in our study. This result is very different from what has been published by other authors. Mirzatolooei et al. ^21^ reported persistence of sensory disturbance in 48.9% of the knees, also in a six-month follow-up. With the same follow-up time, Sabat et al. ^9^ and Sipahioglu et al. ^11^ reported that 32.4% and 42.8% of their patients’ knees continued to have sensory alterations, respectively. For Joshi et al. ^22^ , 11.2% of the knees continued to exhibit sensory changes after 12 months. In a study by Sharaby et al., with a mean follow-up of 23.7 months, they reported sensory changes in only 5.6% of the evaluated knees. On the other hand, Inderhaug et al., ^23^ at a minimum follow-up of 10 years, reported that 85% of the patients had symptoms related to ACLR injury These authors believed that the sensory deficit was likely to be permanent. Table 4 shows the comparisons between size and angulation of the incision, percentage of patients with altered sensitivity, follow-up period, altered sensitivity area, persistence of altered sensitivity area, and main location of dysesthesia according to different authors. 

**Table 4.** Comparisons between size and angulation of the incision, percentage of patients with altered sensitivity, follow-up period, altered sensitivity area, persistence of altered sensitivity area, and main location of dysesthesia according to different authors

Authors and Year of Publication	Incision Length (mm)	Incision Angle (degrees)	Patients with Sensitivity Alteration (%)	Follow-up Time (months)	Area with Sensitivity Alteration (cm²)	Persistence of Sensitivity Alteration in Follow-up	Main Location of Dysesthesia
Luo et al. 2007	33	NI	24.14	14	8.4	NI	NI
Mirzatolooei et al. 2012	32	45°	48.9	6	NI	48.9%	Anterolateral superior
Sabat et al. 2013	38	50°	32.4	6	18.9	32.4%	Inferior portion of incision
Joshi et al. 2016	30	45°	16.6	12	NI	11.2%	Lateral aspect
Leite et al. 2016	30	45°	26	12	NI	NI	Inferior and superior lateral
Sipahioglu et al. 2017	38	50°	45	6	9.3	42.8%	Lateral to TT
Mousavi et al. 2018	38	45°	61.3	6	11.5	NI	NI
Sharaby et al. 2019	NI	NI	41.9	24	NI	5.6%	NI
Keyhani et al. 2020	27	45°	41.6	9	9.6	NI	Anterolateral proximal

MEAN and SD 33.2 ± 4.3 46.4 ± 2.4 37.5 ± 13.9 10.5 ± 5.9 11.5 ± 4.2 16.6 ± 17.2

 As the oblique incision parallel to the IPBSN path for ACLR did not prevent injury in harvesting the hamstring tendons, other factors may have contributed to the occurrence of this condition, such as damage to a secondary branch when performing the AMP and AAMP, as described by Tifford et al. ^24^ and Plancher et al. ^25^ Our findings suggest that we should explore other methods to prevent IPBSN injury. There are some possibilities for this purpose: knowing that the incision should not be performed too medially to the TT, as found in our investigation; identifying the nerve using intraoperative ultrasound ^26^ or during surgery ^21^ ; recognizing the ‘sentinel’ blood vessel adjacent to the insertion of the flexor tendons as a parameter for tendon localization, thereby allowing limited dissection of the wound ^27^ ; releasing the sartorius fascia before harvesting ^28^ ; performing tendon removal via the popliteal access ^28^^,^^30^ ; performing a horizontal incision when creating the AMPP. ^25^ ; and flexing the knee to 110° to create the AAMP. ^25^

There are several limitations to our research: we did not aim to identify the IPBSN during the removal of the flexor tendons; the altered sensitivity area was delineated by the limits of the perceptual change reported by the patients, and these assessments were always performed by only one researcher, not by two members of the team. Although performed by the same person, it was not possible to control the pressure exerted at the time of the test, which may have been different between the knees examined. We only assessed tactile sensitivity; thus, thermal and pain sensitivity were not tested, as well as tactile two-point discrimination. Finally, incision angle and photographed area measurements were conducted by a single researcher; however, ideally, it should have been measured by two authors.

## CONCLUSIONS

The oblique incision parallel to the IPBSN path for ACLR did not prevent injury in harvesting the hamstring tendons, especially in women. However, this condition rarely compromised the activities of daily living.
